# An interpretable machine learning model based on computed tomography radiomics for predicting programmed death ligand 1 expression status in gastric cancer

**DOI:** 10.1186/s40644-025-00855-3

**Published:** 2025-03-12

**Authors:** Lihuan Dai, Jinxue Yin, Xin Xin, Chun Yao, Yongfang Tang, Xiaohong Xia, Yuanlin Chen, Shuying Lai, Guoliang Lu, Jie Huang, Purong Zhang, Jiansheng Li, Xiangguang Chen, Xi Zhong

**Affiliations:** 1 Department of Medical Imaging, Guangzhou Institute of Cancer Research, the Affiliated Cancer Hospital, Guangzhou Medical University, Guangzhou, 510095 China; 2 Department of Radiology, Meizhou People’s Hospital, Mei Zhou, 514031 China; 3 Department of Pathology, Guangzhou Institute of Cancer Research, the Affiliated Cancer Hospital, Guangzhou Medical University, Guangzhou, 510095 China

**Keywords:** Gastric cancer, Programmed death ligand 1, Computed tomography, Radiomics, Machine learning, SHapley additive explanations

## Abstract

**Background:**

Programmed death ligand 1 (PD-L1) expression status, closely related to immunotherapy outcomes, is a reliable biomarker for screening patients who may benefit from immunotherapy. Here, we developed and validated an interpretable machine learning (ML) model based on contrast-enhanced computed tomography (CECT) radiomics for preoperatively predicting PD-L1 expression status in patients with gastric cancer (GC).

**Methods:**

We retrospectively recruited 285 GC patients who underwent CECT and PD-L1 detection from two medical centers. A PD-L1 combined positive score (CPS) of ≥ 5 was considered to indicate a high PD-L1 expression status. Patients from center 1 were divided into training (*n* = 143) and validation sets (*n* = 62), and patients from center 2 were considered a test set (*n* = 80). Radiomics features were extracted from venous-phase CT images. After feature reduction and selection, 11 ML algorithms were employed to develop predictive models, and their performance in predicting PD-L1 expression status was evaluated using areas under receiver operating characteristic curves (AUCs). SHapley Additive exPlanations (SHAP) were used to interpret the optimal model and visualize the decision-making process for a single individual.

**Results:**

Nine features significantly associated with PD-L1 expression status were ultimately selected to construct the predictive model. The light gradient-boosting machine (LGBM) model demonstrated the best performance for PD-L1 high expression status prediction in the training, validation, and test sets, with AUCs of 0.841(95% CI: 0.773, 0.908), 0.834 (95% CI:0.729, 0.939), and 0.822 (95% CI: 0.718, 0.926), respectively. The SHAP summary and bar plots illustrated that a feature’s value affected the feature’s impact attributed to the model. The SHAP waterfall plots were used to visualize the decision-making process for a single individual.

**Conclusion:**

Our CT radiomics–based LGBM model may aid in preoperatively predicting PD-L1 expression status in GC patients, and the SHAP method may improve the interpretability of this model.

**Supplementary Information:**

The online version contains supplementary material available at 10.1186/s40644-025-00855-3.

## Background

Gastric cancer (GC) is the fifth most common malignancy and the fourth leading cause of cancer-related death worldwide [1]. GC typically has a poor prognosis because it is often diagnosed at an advanced stage [2]. Despite several recent advancements in relevant surgical techniques, neoadjuvant chemotherapy, and targeted therapy, GC prognosis has remained poor, with age-standardized 5-year net survival remaining at 20–40% [3]. Therefore, novel, effective treatment strategies for GC applicable in clinical practice are warranted.

Immunotherapy with immune checkpoint inhibitors targeting programmed death ligand 1 (PD-L1)/programmed cell death protein 1 (PD-1) has great applicability in treating various cancers, including GC, melanoma, renal cell carcinoma, and lung cancer [4–9]. However, the immunotherapeutic response rate remains relatively low; thus, selecting patients who may benefit from anti-PD-1/PD-L1 therapy precisely is essential [10]. Tumor PD-L1 expression status is closely associated with the effectiveness of anti-PD-1/PD-L1 immunotherapy, and it is widely used as a feasible molecular biomarker for treatment efficacy prediction [11]. Currently, immunohistochemistry (IHC) is the method most commonly used to evaluate PD-L1 expression status in GC; however, the tissue used for PD-L1 detection is derived from operations or endoscopic tissue biopsy. Tissue-based biopsy is a relatively expensive, invasive procedure associated with varying degrees of harm to the patient [12]. Moreover, if a biopsied tumor tissue is insufficient, precisely determining PD-L1 expression can be difficult because of tumor heterogeneity [13]. Therefore, accurate, noninvasive assessment of PD-L1 expression status is crucial to guiding treatment strategies.

Radiomics, a noninvasive technique for extracting high-dimensional quantitative data from medical images [14, 15], can reflect tumor heterogeneity and provide valuable insights into cancer diagnosis, prognosis, and individualized treatment [16–18]. Studies have indicated that the CT radiomics model with traditional logistic regression (LR) analysis may quantitatively predict PD-L1 expression in several cancers including GC. However, the performance of CT radiomics models reported in these studies remains unclear [19–21].

Machine learning (ML) is being increasingly used in medicine because it can process large amounts of data accurately [22, 23]. However, although most studies have focused on improving the predictive accuracy of ML models, the interpretability of the predictive model remains unclear. Therefore, studies are increasingly focusing on applying interpretable ML models in clinical decision support systems and medical research. Interpretable models allow clinicians to focus on rational decision-making, ensure appropriate model functionality, and guide diagnosis or treatment decisions [24, 25]. Furthermore, rationalizing model decisions aids in prioritizing major outcomes, facilitating the extraction of valuable insights, and enhancing confidence and acceptability of predictions related to PD-L1 expression.

Traditional ML often lacks interpretability, which leads to the “black box” problem, making it unconducive to clinical application. The SHapley Additive exPlanations (SHAP), a method used for addressing an ML model’s interpretability, can illustrate the effects of features on the overall predictive model and visualize the decision-making process for each patient. Recently, the SHAP method was successfully applied to explain various ML models, such as disease and therapeutic prognosis models [26–29]. To our knowledge, this method for ML model interpretation has not been used in predicting PD-L1 expression status thus far. Therefore, here, we developed and validated 11 ML models based on CT radiomics for predicting PD-L1 expression status in GC and used the SHAP method to explain and visualize our models.

## Materials and methods

### Patients

**Fig. 1:**
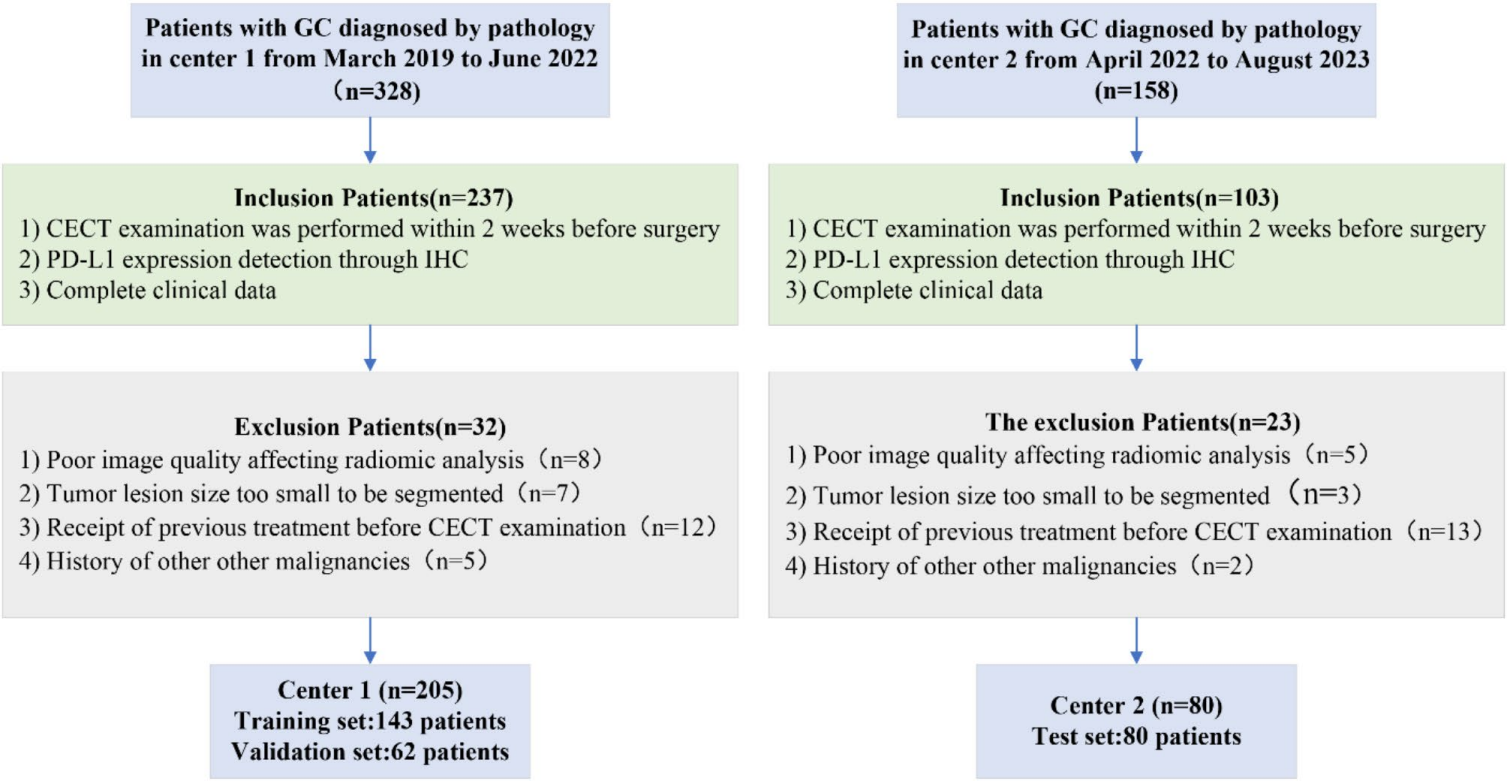
Flow of patient recruitment. PD-L1, programmed death-ligand 1; CECT, contrast-enhanced computed tomography

Figure 1 illustrates the current patient recruitment flow. In this retrospective clinical study, we included data from consecutive patients diagnosed as having pathologically confirmed GC between March 2019 and August 2023 at Affiliated Cancer Hospital & Institute of Guangzhou Medical University (center 1) or Meizhou People’s Hospital (center 2). The inclusion criteria were (1) CECT examination performed within 2 weeks before surgery, (2) PD-L1 expression detection through IHC, and (3) complete clinical data. The exclusion criteria were (1) poor image quality affecting radiomics analyses, (2) tumor lesion size too small to be segmented, (3) receipt of previous treatment before CECT examination, and (4) history of other malignancies. Finally, 205 center-1 patients (129 men and 76 women) aged 27–83 years (median age, 59 years) were randomly divided into training and validation sets at a ratio of 7:3. Moreover, 80 center-2 patients (50 men and 30 women) aged 27–79 years (median age, 57 years) were included in the test set. The following clinical data were retrieved for each patient: sex, age, serum tumor markers [carcinoembryonic antigen (CEA), carbohydrate antigen 19 − 9 (CA19-9), carbohydrate antigen 24 − 2 (CA24-2), and carbohydrate antigen 72 − 4 (CA72-4)], and TNM stage (AJCC, 8th edition). The threshold values for CEA, CA19-9, CA24-2, and CA72-4 levels were set at 5.0 µg/mL, 30 U/mL, 20 U/mL, and 6.9 U/mL, respectively.

This study was approved by the Ethics Committee of Affiliated Cancer Hospital & Institute of Guangzhou Medical University, and the requirement for informed consent was waived considering the design of this study.

### PD-L1 detection and expression classification

For PD-L1 expression detection, GC tumor tissue sections were subjected to standard IHC staining with a PD-L1 IHC 22C3 pharmDx assay kit (Agilent Technologies). PD-L1 expression was quantified based on the combined positive score (CPS), which was calculated as follows: CPS = [(PD-L1 membrane staining positive tumor cells + PD-L1 membrane staining positive tumor-associated immune cells)/Total number of tumor cells] × 100. The immunostained tissue sections were scored by two independent pathologists (X.H.X. and Y.L.C. with 10 and 6 years of relevant experience, respectively). Both pathologists were blinded to the patients’ clinical data, and disagreements on CPS assessment were resolved through consensus 2 weeks after individual interpretations. CPSs of ≥ 5 and < 5 were considered to indicate PD-L1 high and low expression statuses, respectively [4, 6, 30]. Supplementary A1 presents additional details regarding the PD-L1 detection method and expression classification.

### CECT image acquisition

Table S1 presents the CT scanners and image acquisition protocols at centers 1 and 2. After an unenhanced CT scan, all patients were injected with a nonionic iodinated contrast medium (Ioversol 320 iodine/mL from Jiangsu Hengrui Medicine or Ultravist 370 from Bayer Schering Pharma) at a dose of 1.5 mL/kg and an injection rate of 3 mL/s with a high-pressure pump syringe. Arterial and venous-phase images were taken at 25 and 65 s after contrast agent injection, respectively.

### Image segmentation and radiomics feature extraction

Two radiologists—named reader 1 (J.X.Y.) and reader 2 (Y.F.T.) with 9 and 5 years of image processing experience, respectively—segmented the regions of interest (ROIs) by manually delineating GC lesion boundaries on each venous-phase image section depicting the maximum tumor area. First, reader 1 segmented the ROIs using ITK-SNAP (version 3.60; http://www.itksnap.org). After 1 month, 30 patients were randomly selected and resegmented by readers 1 and 2, and the intraobserver and interobserver agreements were assessed. Radiomics features were extracted using the Pyradiomics package of Pyradiomics (version 3.1.0; https://pypi.org/project/pyradiomicsss/). To eliminate differences in image resolution and pixel size generated by different CT equipment, all CT images were resampled to a voxel spacing of 1 × 1 × 1 mm and discretized with a bin width of 25 HU before feature extraction. Table S2 lists the details of the obtained radiomics features.

### Radiomics feature selection

Before feature selection, the radiomics features were standardized using the Z-score method. Four steps were performed for dimensionality reduction and selection of radiomics features in the training set: (1) Features with interclass and intraclass correlation coefficients > 0.75 were retained. (2) Features with correlation coefficient > 0.9 were considered highly correlated, and one of every two features was discarded for redundancy with the other feature. (3) Univariate analysis was used to select features significantly associated with PD-L1 expression status, and features with *p* < 0.05 were reserved. (4) The least absolute shrinkage and selection operator (LASSO) regression with 10-fold cross-validation was used to select the most relevant features.

### ML model construction and interpretation

**Fig. 2:**
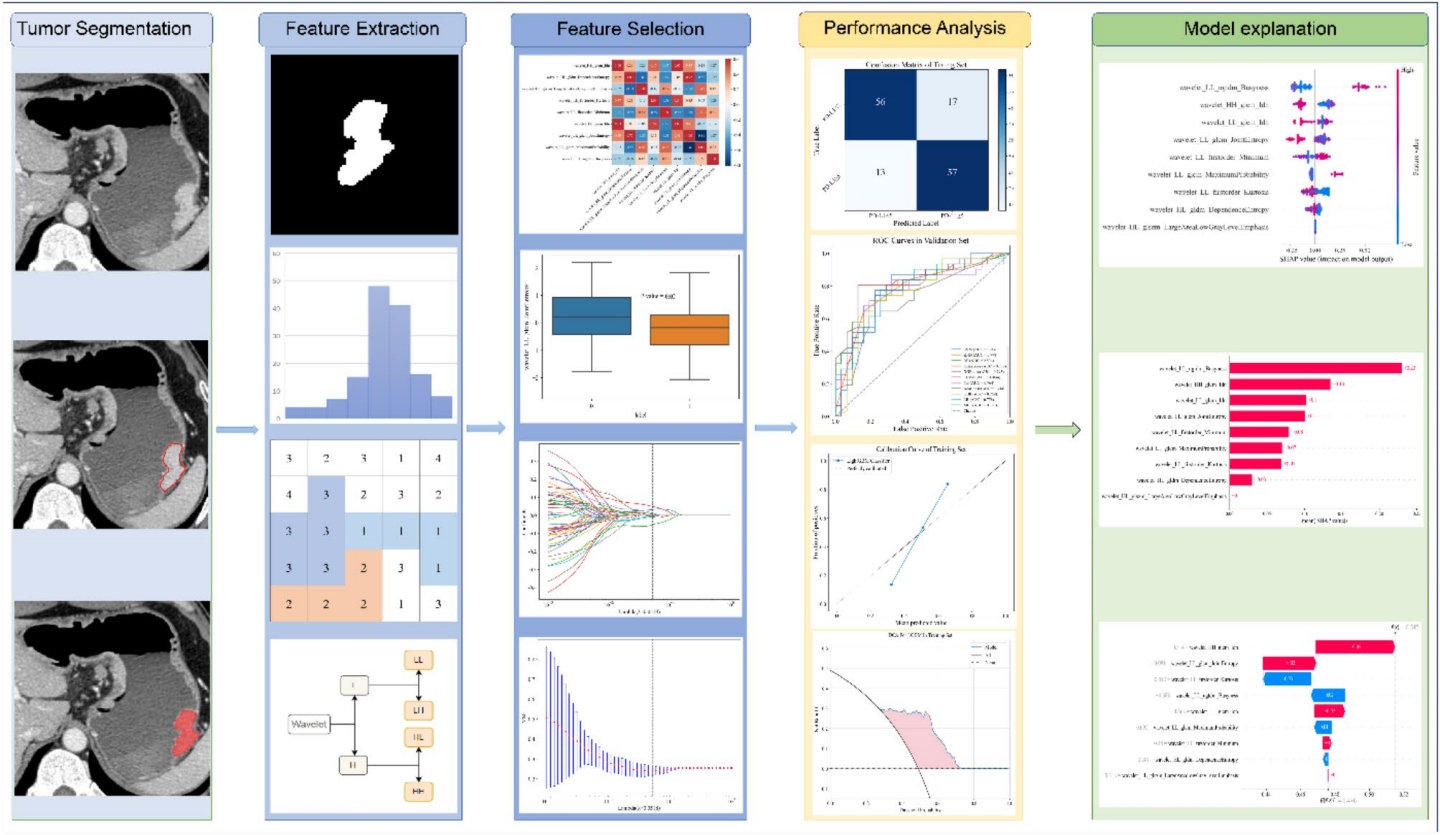
Current study workflow

To select the optimal model for predicting PD-L1 expression status in patients with GC, 11 mainstream algorithms were selected to build models in the training set: LR, naïve Bayes (NB), support vector machine (SVM), K-nearest neighbors (KNNs), random forest (RF), extremely randomized trees (ExtraTrees), extreme gradient boosting (XGBoost), light gradient-boosting machine (LGBM), gradient-boosting regression (GBR), adaptive boosting (AdaBoost), and multilayer perceptron (MLP). The model with the highest area under the receiver operating characteristic (ROC) curve (AUC) in the validation set was considered the optimal model. The SHAP method was used to improve the optimal model’s interpretability. SHAP summary and bar plots were drawn to illustrate the features’ importance and visualize their impacts on the model with SHAP values. Furthermore, the SHAP waterfall plots were used to explore individual-based decision-making processes from a local explanation perspective. Figure 2 illustrates the workflow of this study.

### Statistical analyses

Statistical analysis was performed using R (version 4.12; https://www.r-project.org/) and Python (version 3.913; https://www.python.org/). A *p-*value of < 0.05 was considered to indicate statistical significance. Categorical variables, compared with the chi-square or Fisher’s exact test, are expressed as ratios (percentages). The Kolmogorov–Smirnov test was performed to test the normal distribution of continuous quantitative data. Continuous data are presented as means ± standard deviation (SD) or median (Q₁, Q₃). We compared normally and nonnormally distributed continuous data by using an independent *t*-test and the Mann–Whitney *U* test, respectively. The performances of each ML model were evaluated by using ROC analysis, and the AUC, accuracy, sensitivity, specificity, positive predictive value (PPV), negative predictive value (NPV), precision, recall, and F1 score were calculated. Calibration curves were used to evaluate the agreement between the predicted and postoperative pathological IHC results in the training, validation, and test sets. The decision curve analysis (DCA) was performed to reveal the clinical utility of our ML models.

## Results

### Patient characteristics

Of all 285 patients included in this study, 143 (50.18%), 62 (21.75%), and 80 (28.07%) were assigned to the training, validation, and test sets, respectively. In total, 70 (48.95%), 31 (50.00%), and 34 (42.50%) patients in the training, validation, and test sets demonstrated PD-L1 high expression status, respectively. The baseline characteristics, including PD-L1 expression status, sex, age, serum tumor markers (CEA, CA199, CA142, and CA724), and TNM classification, did not differ significantly between the training, validation, and test sets. Table 1 summarizes the included patients’ clinical characteristics.

**Table 1:** Patient clinical characteristics in training, validation, and test sets

Characteristic	Training set (*n* = 143)	Validation set (*n* = 62)	Test set (*n* = 80)	*P* value
**PD-L1 expression (No. %)**				0.584
CPS < 5	73 (51.1%)	31 (50.0%)	46 (57.5%)	
CPS ≥ 5	70 (49.0%)	31 (50.0%)	34 (42.5%)	
Age* (years)	59.0 (29–83)	59.0 (27–81)	57.0 (27–79)	0.563
**Sex (No. %)**				0.636
Male	50 (35.0%)	26 (41.9%)	30 (37.5%)	
Female	93 (65.0%)	36 (58.1)	50 (62.5%)	
**CEA (No. %)**				0.084
< 5.0 µg/ml	103 (72.0%)	40 (64.5%)	46 (57.5%)	
≥ 5.0 µg/ml	40 (28.0%)	22 (35.5%)	34 (42.5%)	
**CA199 (No. %)**				0.314
< 30 U/mL	108 (75.5%)	46 (74.2%)	53 (66.3%)	
≥ 30 U/mL	35 (24.5%)	16 (25.8%)	27 (33.7%)	
**CA242 (No. %)**				0.107
< 20 U/mL	116 (81.1%)	51 (82.3%)	56 (70.0%)	
≥ 20 U/mL	27 (18.9%)	11 (17.7%)	24 (30.0%)	
**CA724 (No. %)**				0.304
< 6.9 U/mL	101 (70.6%)	44 (71.0%)	49 (61.3%)	
≥ 6.9 U/mL	42 (29.4%)	18 (29.0%)	31 (38.7%)	
**TNM stage (No. %)**				0.488
I	14 (9.8%)	9 (14.5%)	11 (13.8%)	
II	34 (23.8%)	19 (30.7%)	25 (31.3%)	
III	86 (60.1)	29 (46.8%)	37 (46.2%)	
IV	9 (6.3%)	5 (8.1%)	7 (8.7%)	

Note: Presented as No. %, and compared using the chi-square or Fisher’s exact test. ^*^Presented as median (range), and compared using the Mann–Whitney *U* test

Abbreviations: PD-L1, programmed death-ligand 1; CPS, combined positive score; CEA, carcinoma embryonic antigen; CA199, carbohydrate antigen 19 − 9; CA72, carbohydrate antigen 72 − 4; CA242, carbohydrate antigen 24 − 2; TNM, tumor node metastasis

### Radiomics feature selection

**Fig. 3:**
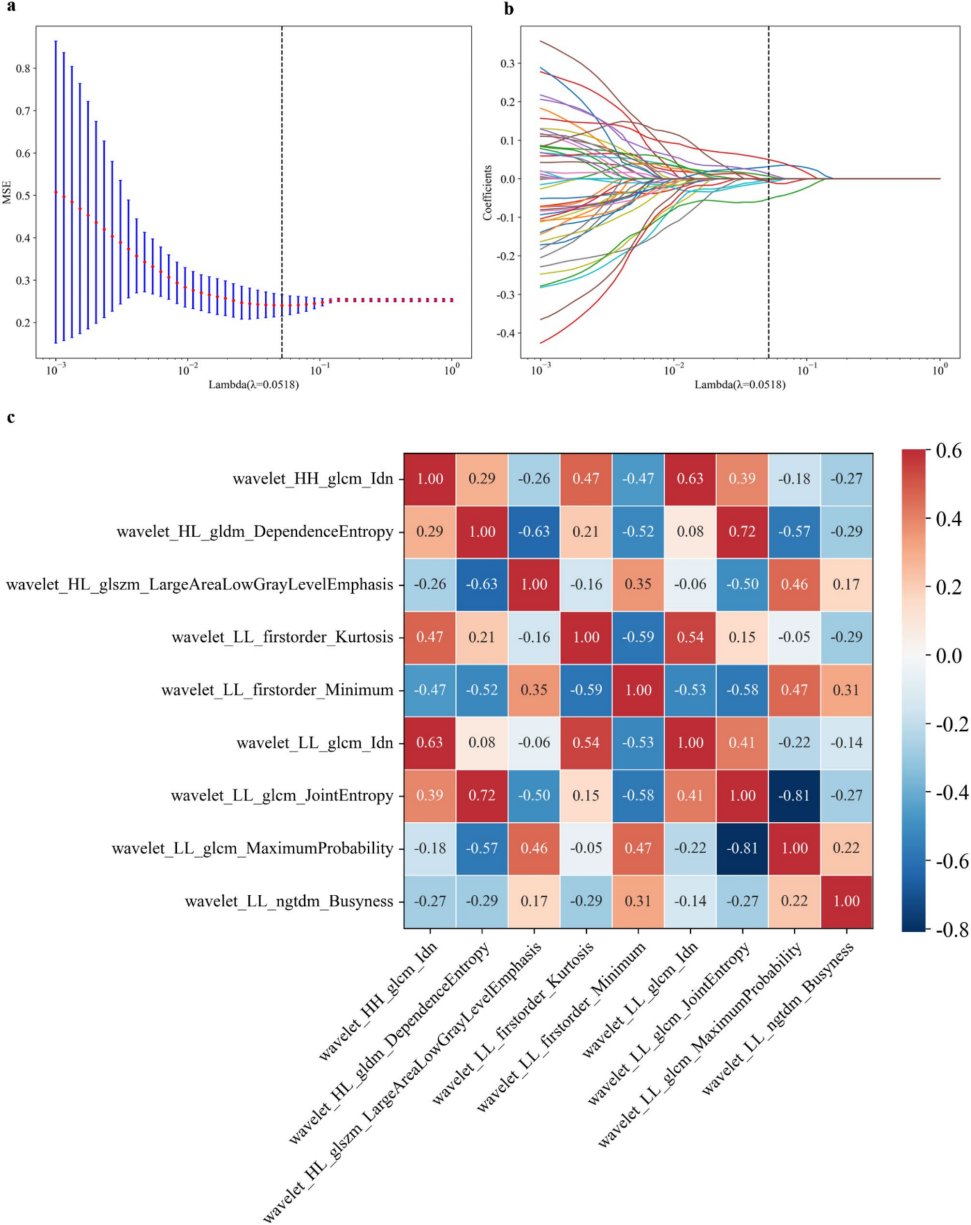
Radiomics feature selection using least absolute shrinkage and selection operator (LASSO) regression and features correlation heatmaps. (**a)** Tuning parameter selection (λ) in the LASSO model via 10-fold cross-validation based on minimum criteria. Optimal values of the LASSO tuning parameter (λ) are indicated using dotted vertical lines. A λ value of 0.0518 was selected. (**b)** When λ = 0.0518, LASSO regression reduced the number of features to nine. (**C)** Correlation heatmap of the nine radiomics features in the training set

From each ROI, we extracted 476 radiomics features, of which 366 features with interclass and intraclass correlation coefficients ≥ 0.75 were selected for further reduction. After Pearson or Spearman correlation analyses, 130 features were retained. The independent-sample *t-*test or Mann–Whitney *U* test revealed 46 features with significant differences between PD-L1 high and low expression groups in the training set. The LASSO LR model was used to reduce the number of features from 46 to 9, with an optimal regulation weight (λ) of 0.0518 under the minimum criterion (Fig. 3a, b). Figure 3c presents the correlation heatmap of these nine features. A comparison of the selected features’ names and values between PD-L1 high and low expression groups in the training set is presented in Table 2; similar comparison results for the validation and test sets are detailed in Table S3. Fig. S2a and b illustrate the correlation heatmaps of these nine features in the validation and test sets, respectively. Table S4 lists the ICC coefficients for interobserver and intraobserver repeatability of radiomics features included in the final model.

**Table 2:** Comparison of radiomics features between PD-L1 high and low expression groups in training set

Features	Radiomics feature value (Z-score normalization)		
	PD-L1 low Expression (*n* = 73)	PD-L1 high Expression (*n* = 70)	*P* value
wavele_HH_glcm_Idn	0.25 ± 0.99	-0.26 ± 0.95	0.002
wavele_HL_gldm_Dependence-Entropy	0.21 ± 0.98	-0.22 ± 0.97	0.009
wavele_HL_glszm_LargeAreaLowGrayLevelEmphasis	-0.18 ± 0.70	0.19 ± 1.21	0.005
wavele_LL_firstorder_Kurtosis	0.25 ± 1.24	-0.26 ± 0.56	0.009
wavele_LL_firstorder_Minimum	-0.29 ± 1.04	0.30 ± 0.87	< 0.001
wavele_LL_glcm_Idn	0.28 ± 0.96	-0.29 ± 0.97	< 0.001
wavele_LL_glcm_JointEntropy	0.25 ± 0.95	-0.27 ± 0.99	0.002
wavele_LL_glcm_Maximum-Probability	-0.22 ± 0.77	0.23 ± 1.16	0.015
wavele_LL_ngtdm_Busyness	-0.25 ± 0.73	0.26 ± 1.17	0.009

Note: Data are presented as mean ± standard deviation, and compared using an independent *t-*test

Abbreviations: CPS, combined positive score; glcm, gray level cooccurrence matrix; glszm, gray level size zone matrix; ngtdm, neighboring gray tone difference matrix

### Predictive performance of ML models

**Fig. 4:**
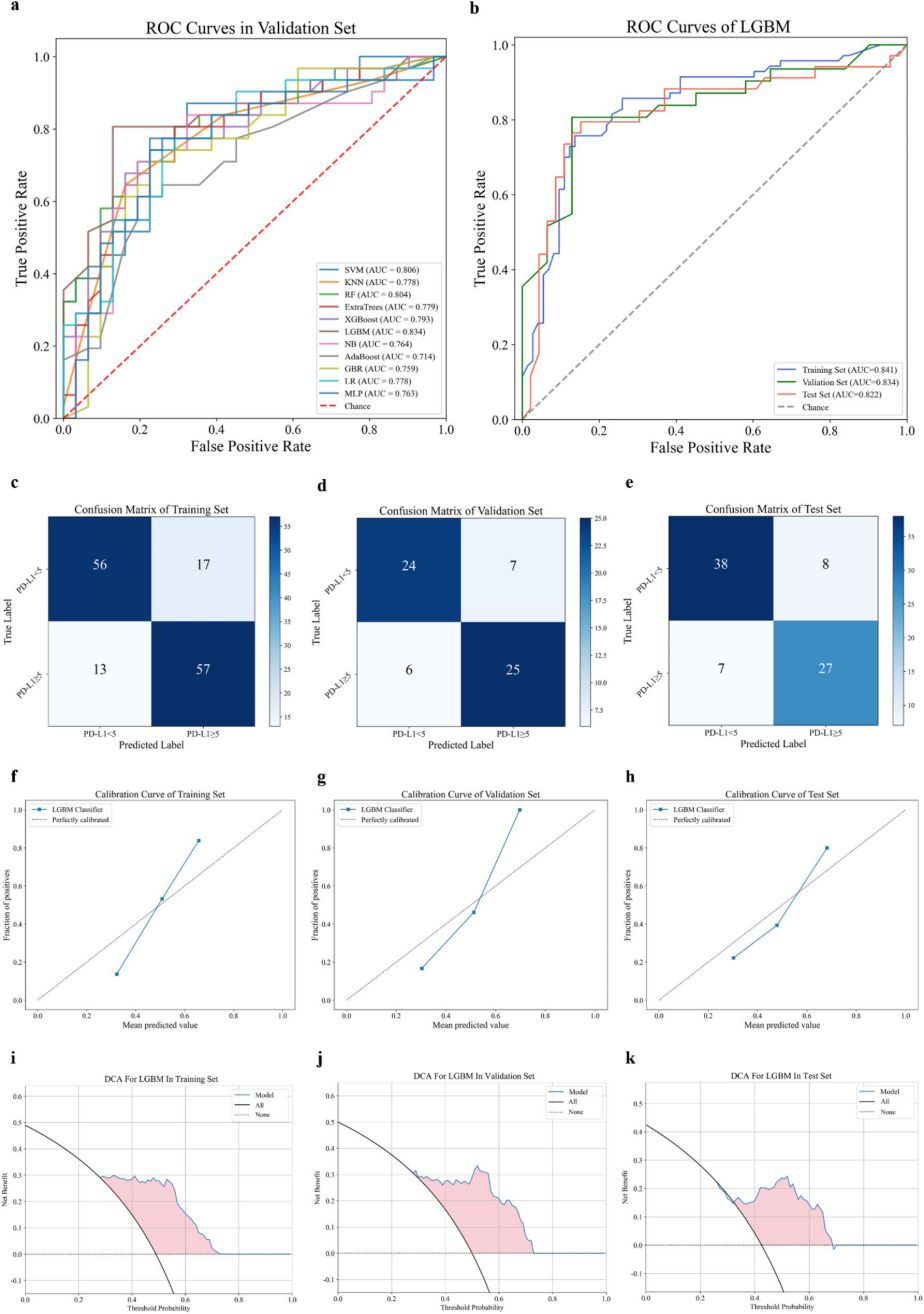
Prediction performance of ML models. (**a)** ROC curves of 11 ML models in the validation set. (**b**) ROC curves of the LGBM model in the training, validation, and test sets (**c**-**e**) Confusion matrices for the (**c**) training, (**d**) validation, and (**e**) test sets. (**f**-**h**) Calibration curves for the (**f**) training, (**g**) validation, and (*h*) test sets. (**i**-**k**) Decision curves for the (**i**) training, (**j**) validation, and (**k**) test sets. ML, machine learning; LGBM, light gradient boosting machine; ROC, receiver operating characteristic

As presented in Table 3, all 11 predictive models demonstrated good performance in classifying PD-L1 high expression status from PD-L1 low expression status in all sets; the AUC values were 0.734–0.961, 0.763–0.834, and 0.686–0.822 in the training, validation, and test sets, respectively. Of all 11 ML models, the LGBM model achieved the highest AUC of 0.834 (95% CI: 0.729, 0.939) in the validation set (Fig. 4a) and was thus identified as the optimal model for predicting PD-L1 expression status. Figure 4b presents the ROC curves of the LGBM model for the training, validation, and test sets. The confusion matrices of the LGBM model in the training, validation, and test sets revealed that the model accurately detected GC patients with high PD-L1 expression status (sensitivity: 0.743, 0.774, and 0.765, respectively) and effectively differentiate between patients with low PD-L1 expression status (specificity: 0.863, 0.871, and 0.848, respectively; Fig. 4c-e). The LGBM model calibration curve also demonstrated good agreement between the predicted and postoperative pathological IHC results in all sets (Fig. 4f-h). The DCA curves revealed that the LGBM model had overall net benefits for predicting PD-L1 expression status, with the majority of the range of reasonable threshold probabilities in all sets (Fig. 4i-k). Fig. S2 displays the ROC curves for all 11 models in the training and test sets.

**Table 3:** Performance of 11 ML models for PD-L1 expression status prediction

Model	Set	AUC (95% CI)	Accuracy	Sensitivity	Specificity	PPV	NPV	Precision	Recall	F1 Score
**SVM**	Training set	0.812 (0.739–0.885)	0.755	0.743	0.767	0.754	0.757	0.754	0.743	0.748
	Validation set	0.806 (0.698–0.915)	0.758	0.742	0.774	0.767	0.750	0.767	0.742	0.754
	Test set	0.768 (0.663–0.873)	0.725	0.500	0.891	0.773	0.707	0.773	0.500	0.607
**KNN**	Training set	0.791 (0.722–0.862)	0.664	0.386	0.932	0.844	0.613	0.844	0.386	0.529
	Validation set	0.778 (0.661–0.895)	0.613	0.29	0.935	0.818	0.569	0.818	0.290	0.429
	Test set	0.686 (0.567–0.805)	0.663	0.588	0.717	0.606	0.702	0.606	0.588	0.597
**RF**	Training set	0.914 (0.868–0.960)	0.853	0.814	0.890	0.877	0.833	0.877	0.814	0.844
	Validation set	0.804 (0.693–0.915)	0.742	0.774	0.710	0.727	0.759	0.727	0.774	0.750
	Test set	0.754 (0.643–0.865)	0.750	0.559	0.891	0.792	0.732	0.792	0.559	0.655
**ExtraTrees**	Training set	0.807 (0.735–0.879)	0.762	0.714	0.808	0.781	0.747	0.781	0.714	0.746
	Validation set	0.779 (0.661–0.897)	0.742	0.774	0.710	0.727	0.759	0.727	0.774	0.750
	Test set	0.740 (0.624–0.856)	0.750	0.559	0.891	0.792	0.732	0.792	0.559	0.655
**XGBoost**	Training set	0.961 (0.933–0.989)	0.909	0.900	0.918	0.913	0.905	0.913	0.900	0.906
	Validation set	0.793 (0.680–0.907)	0.742	0.645	0.839	0.800	0.703	0.800	0.645	0.714
	Test set	0.782 (0.675–0.889)	0.763	0.618	0.870	0.778	0.755	0.778	0.618	0.689
**LGBM**	Training set	0.841 (0.773–0.908)	0.804	0.743	0.863	0.839	0.778	0.839	0.743	0.788
	Validation set	0.834 (0.729–0.939)	0.823	0.774	0.871	0.857	0.794	0.857	0.774	0.814
	Test set	0.822 (0.718–0.926)	0.813	0.765	0.848	0.788	0.83	0.788	0.765	0.776
**NB**	Training set	0.737 (0.654–0.820)	0.727	0.786	0.671	0.696	0.766	0.696	0.786	0.738
	Validation set	0.764 (0.638–0.890	0.742	0.806	0.677	0.714	0.778	0.714	0.806	0.758
	Test set	0.780 (0.674–0.887)	0.738	0.735	0.739	0.676	0.791	0.676	0.735	0.704
**AdaBoost**	Training set	0.875 (0.819–0.931)	0.797	0.943	0.658	0.725	0.923	0.725	0.943	0.820
	Validation set	0.714 (0.584–0.844)	0.694	0.581	0.806	0.75	0.658	0.750	0.581	0.655
	Test set	0.748 (0.636–0.861)	0.725	0.471	0.913	0.800	0.700	0.800	0.471	0.593
**GBR**	Training set	0.936 (0.898–0.975)	0.874	0.886	0.863	0.861	0.887	0.861	0.886	0.873
	Validation set	0.759 (0.635–0.883)	0.726	0.677	0.774	0.750	0.706	0.750	0.677	0.712
	Test set	0.731 (0.619–0.843)	0.713	0.647	0.761	0.667	0.745	0.667	0.647	0.657
**LR**	Training set	0.734 (0.650–0.817)	0.720	0.743	0.699	0.703	0.739	0.703	0.743	0.722
	Validation set	0.778 (0.661–0.896)	0.726	0.710	0.742	0.733	0.719	0.733	0.710	0.721
	Test set	0.789 (0.686–0.892)	0.713	0.882	0.587	0.612	0.871	0.612	0.882	0.723
**MLP**	Training set	0.750 (0.669–0.832)	0.741	0.743	0.740	0.732	0.75	0.732	0.743	0.738
	Validation set	0.763 (0.637–0.888)	0.742	0.710	0.774	0.759	0.727	0.759	0.710	0.733
	Test set	0.772 (0.665–0.880)	0.750	0.706	0.783	0.706	0.783	0.706	0.706	0.706

Abbreviations: AUC, area under the receiver operating characteristic curve; CI, confidence interval; NPV, negative prediction value; PPV, positive predictive value; LR, logistic regression; NB, naïve Bayes; SVM, support vector machine; KNN, K-nearest neighbor; RF, random forest; ExtraTrees, extremely randomized trees; XGBoost, extreme gradient boosting; LGBM, light gradient boosting machine; GBR, gradient boosting regression; AdaBoost, adaptive boosting; MLP, multilayer perceptron

### Model interpretation

**Fig. 5:**
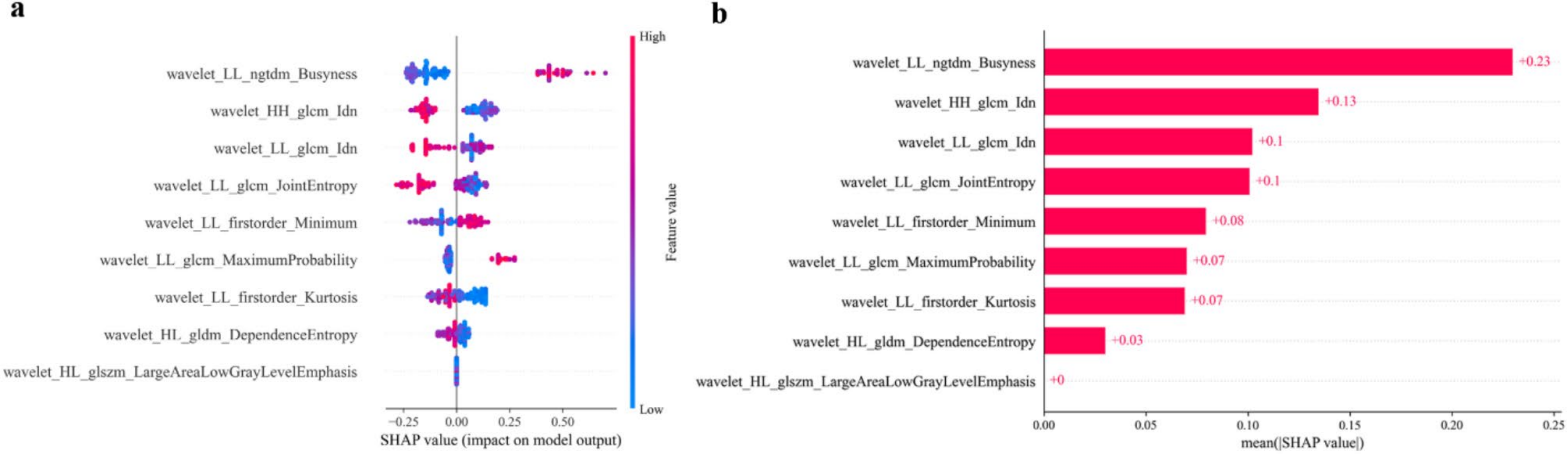
SHAP summary and bar plots. (**a**) SHAP summary plots demonstrating the distribution of effects of each feature on the LGBM model outputs. Red and blue denote high and low feature values, respectively. The *x*-axis represents the effects of the SHAP values on the model output. The larger the value on the x-axis, the greater was the probability of PD-L1 high expression. (**b**) SHAP bar plot displaying the distribution of importance of nine features in the LGBM model. The value to the right of each red bar is the contribution coefficient of the feature to the model, which is the absolute value of the average of the SHAP value of each feature. SHAP, Shapley additive explanation; LGBM, light gradient boosting machine; PD-L1, programmed death ligand 1

The SHAP method was used to obtain quantitative explanation for the LGBM model. In the global visualization, we drew a SHAP summary plot (Fig. 5a), indicating the relationship between each feature’s value and its impact on the model, as well as the positive or negative effects of each feature on the prediction probability. We also drew a SHAP bar plot (Fig. 5b), which demonstrated the mean of the absolute average SHAP values for the nine radiomics features. The top four influential features were wavelet_LL_ngtdm_Busyness, wavelet_HH_glcm_Idn, wavelet_LL_glcm_Idn, and wavelet_LL glcm_JointEntropy, with absolute average SHAP values of 0.23, 0.13, 0.1, and 0.1, respectively.

**Fig. 6:**
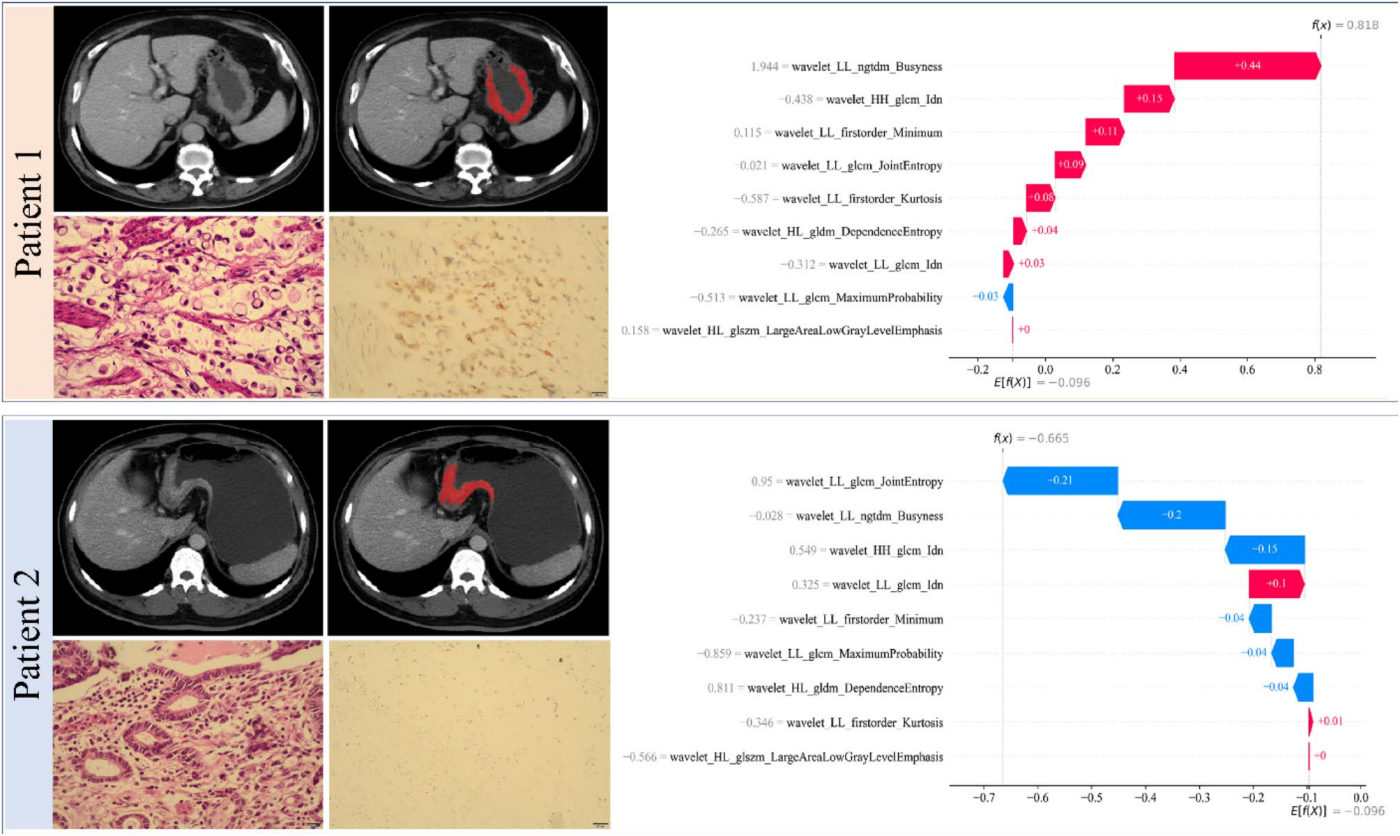
Individual visualization of the mode through SHAP. Patients 1 and 2 are examples of correctly predicted PD-L1 high (CPS = 8) and low (CPS = 0) expression cases, respectively. Legend for each patient shows CT images of GC in the venous stage, manual tumor segmentation, hematoxylin–eosin-stained sections, Immunohistochemistry image presenting PD-L1 expression (magnification: 200×), and the SHAP waterfall plot. SHAP, Shapley additive explanation; PD-L1, programmed death ligand 1; CPS, combined positive score

In the local visualization, Fig. 6 displays two typical examples of correctly predicted PD-L1 high and low expression. Our SHAP waterfall plot was drawn to demonstrate the impact of each feature on the prediction, with the red and blue bars indicating positive and negative impacts, respectively. The base value (E[f(x)]) represents the average SHAP value across all predictions, whereas f(x) represents the final SHAP value. For patient 1, the final SHAP value of 0.818 was larger than the base value (− 0.096), indicating that the model accurately classified this patient into the PD-L1 high expression group, and the feature with the highest contribution was wavelet_LL_ngtdmt_Busyness, with SHAP value = 0.44. In contrast, for patient 2, the final SHAP value of − 0.665 was lower than the base value (− 0.096), suggesting that this patient was accurately categorized into the PD-L1 low expression group. The Wavelet_LL_glcm_JointEntropy demonstrated the greatest negative impact on the prediction outcome, with SHAP value = − 0.21.

## Discussion

In the present study, we developed and validated 11 ML models based on CECT radiomics to predict PD-L1 expression status in patients with GC. All our models demonstrate potential for predicting PD-L1 expression status. Nevertheless, our LGBM model demonstrated the best performance with AUCs of 0.841, 0.834, and 0.822 in the training, validation, and test sets, respectively. Furthermore, by using the SHAP method, we enhanced the interpretability of this model. In general, these results demonstrated that our interpretable LGBM model based on CECT radiomics may provide a noninvasive, reliable method for predicting PD-L1 expression status in patients with GC.

In this study, we used wavelet transform to enhance image contrast, improve edge detection, and reduce noise. As such, we could extract wavelet features better representing the image texture and capture tumor heterogeneity more accurately [31]. Here, we identified nine wavelet-transformed features significantly associated with PD-L1 expression status in GC. For instance, Zheng et al. [20] developed a model comprising nine CT radiomics features to predict PD-L1 expression in head and neck squamous cell carcinoma; of them, seven features were wavelet-transformed features. The authors reported AUCs of 0.852 and 0.802 in the training and validation sets, respectively. Similarly, Jiang et al. [21] demonstrated that eight (88.9%) of nine selected features for a CT-based radiomics signature were wavelet-transformed features. The signature yielded an AUC of 0.96 for the prediction of PD-L1 expression status in non–small-cell lung cancer. The aforementioned findings are consistent with our results: the radiomics features significantly associated with PD-L1 expression in our ML models were all derived from wavelet transformation. Here, wavelet_LL_ngtdm_Busyness, in which wavelet transform analysis of low-frequency components (LL) were integrated with texture characterization via the neighborhood gray-tone difference matrix (NGTDM), demonstrated the highest predictive contribution in our LGBM model. This hybrid approach captured multiscale spatial information through frequency decomposition (distinguishing high-frequency edges from low-frequency morphology) and quantified textural complexity via NGTDM-derived busyness—a metric reflecting local intensity variations. These factors may be associated with tumor heterogeneity and tumor immune microenvironment changes associated with PD-L1 expression status.

Studies have highlighted the potential of CT radiomics as a noninvasive method for predicting PD-L1 expression status in patients with GC [19, 32], but these predictive models were built with a single ML algorithm and did not compare the performance of different ML algorithms. In contrast to these studies, we systematically incorporated 11 ML algorithms to construct predictive models, each offering distinct methodological advantages: the linear LR provided simplicity and interpretability for linear relationships, the probabilistic NB excelled at high-dimensional tasks such as text classification, SVM leveraged kernel tricks for nonlinear separability in high-dimensional spaces, KNN offered instance-based flexibility for small datasets, RF and ExtraTrees reduced overfitting through randomization, XGBoost and LGBM optimized speed and accuracy for structured data, GBR handled nonlinearity with gradient boosting, AdaBoost focused on hard-to-classify samples, and finally, the highly flexible MLP captured complex patterns in data. These algorithms have been validated in similar medical imaging studies [33, 34]. We also comprehensively compared these ML algorithms to identify the best one to predict PD-L1 expression in GC to guide clinical treatment decisions. We ulitimately found that the LGBM model had the highest prediction performance, yielding AUCs of 0.841 and 0.834 in the training and validation sets, respectively. Second, we included more cases and evaluated LGBM model performance by using independent external data (i.e., test set) and noted that this model also demonstrated good discriminative ability for classifying PD-L1 expression status, with an AUC of 0.822. In particular, studies using an LR model [19] and a deep learning model [32] for predicting PD-L1 expression status in GC reported AUCs of 0.774 and 0.784 in the validation set, respectively. Our LGBM model appeared to improve the prediction efficiency, with an AUC of 0.834 in the validation set. Thus, LGBM may be an optional, effective ML algorithm to classify PD-L1 expression status in patients with GC.

LGBM is a gradient-boosting framework based on tree-based learning algorithms [35], and several studies have demonstrated its favorable predictive value in medicine. For instance, Dong et al. investigated the occurrence of sarcopenia in patients with advanced non–small cell lung cancer by combining CT radiomics features with the LGBM classifier and noted AUCs of 0.940 and 0.889 in the training and validation sets, respectively [36]. Leng et al. developed five ML models based on CT radiomics to preoperatively predict epithelial ovarian cancer stages and demonstrated that the LGBM model had notable prediction efficiency and robustness, yielding AUCs of 0.83, 0.80, and 0.68 in the training, internal validation, and external validation cohorts, respectively [37]. In the current study, LGBM was optimized for computational efficiency through a histogram-based algorithm, which significantly reduced processing time and memory use and prevented overfitting through built-in regularization. As a gradient-enhanced tree model, LGBM could effectively capture the nonlinear relationship between radiomics features. This may be particularly applicable to the identification of tumor heterogeneity and microenvironment changes in CT images, making it more conducive to predicting PD-L1 expression in patients with GC.

Although ML predictive models have been reported to be powerful [33, 38–43], they are often referred to as black boxes because they lack interpretability and transparency [44]. SHAP, a highly practical ML interpretation tool, can open the black box of ML predictive models by providing both global and local explanations in a clinician-friendly manner, promoting the clinical application of models and boosting clinicians’ confidence in using predictive models. Studies have employed SHAP to efficiently interpret and visualize radiomics models developed using various ML algorithms. For instance, Wang et al. [45] found that SHAP summary plot effectively illustrated the value of MRI radiomics features in influencing the impact attributable to the SVM model in assessing responses to whole-brain radiotherapy for brain metastases. Moreover, the SHAP force plot quantified the integration of feature impacts on individual responses through SHAP values. Liu et al. [46] developed an XGBoost combined model for predicting perineural invasion in intrahepatic cholangiocarcinoma by combining clinicoradiological features and CT radiomics. Their SHAP bar chart demonstrated that compared with clinicopathological features, the radiomics score had the optimal contribution with the highest SHAP value of 0.38 (range, 0.25–0.28). Furthermore, their SHAP force plots demonstrated each feature’s positive and negative impacts on predictive outcomes in individual visualizations. In line with these studies, we applied the SHAP method to interpret and visualize our LGBM models. Our SHAP summary plot provided a global explanation of distribution and importance of feature impacts on model outputs and found that among all radiomics features, wavelet_LL_ngtdm_Busyness had the most important weight, with the highest SHAP value of 0.23 (range, 0.03–0.23). After understanding how features impact the LGBM model, clinicians may use our model to assess individual outcomes. To visualize the model’s prediction results and determine the influence of features on the outcome, we used SHAP waterfall plots. By comparing the output SHAP value of a single patient with the base value (− 0.096), clinicians could easily classify the patient into either the PD-L1-high ( ≥ − 0.096) or -low ( < − 0.096) expression status group. Moreover, clinicians could assess how each feature impacted each patient’s assessment by reviewing the arrow’s color (e.g., red indicating an increased probability of PD-L1-high expression status) and length (describing the degree to which a particular feature contributed to the prediction). SHAP waterfall plots significantly improved clinician comprehension of the decision-making process of the predictive model, strengthening confidence in both algorithmic reliability and clinical applicability of predictions.

In summary, this study established a novel multialgorithm framework for predicting PD-L1 expression in GC via CECT radiomics. By systematically evaluating 11 ML models, we identified LGBM as the optimal model, achieving AUCs of 0.834 (validation set) and 0.822 (external test set). Our key innovation is related to the dominance of wavelet-transformed features (e.g., wavelet_LL_ngtdm_Busyness), uniquely capturing multiscale tumor heterogeneity linked to PD-L1-driven immune microenvironment remodeling. After integrating SHAP, we obtained global quantification of feature contributions and individualized decision visualizations, overcoming the “black box” limitations of traditional models. Rigorous external validation and a robust cohort (*n* = 285) underscore our model’s generalizability and reliability. In general, our study provided a noninvasive, reliable method for predicting the PD-L1 expression status of patients with GC.

This study has several limitations. First, although our model demonstrated good predictive performance across all three datasets from two centers, we used a retrospective design, which may have introduced potential bias. Therefore, prospective studies with multicenter datasets are necessary for further validation. Second, as described previously [19, 32], we performed the manual segmentation of GC tumors, which was both time- and labor-intensive. Thus, future studies should focus on developing automatic, reliable segmentation methods that segment using the artificial intelligence–based approach, as reported previously [47]. Third, the heterogeneity of CT scanners and imaging parameters between centers 1 and 2 may have influenced the distribution of radiomics features. To mitigate this issue, we resampled all images from both centers to a uniform size. Finally, although we adopted 5 as the CPS cutoff for PD-L1 high expression, as reported previously [6, 32], the optimal cutoff of high PD-L1 expression in clinical practice for GC remains unclear. Therefore, large-scale, multicenter prospective studies should be conducted to compare the predictive performance of different CPS cutoffs and identify the optimal value for predicting immunotherapeutic responses.

## Conclusion

The ML model based on CECT radiomics can effectively and non-invasively differentiate between PD-L1 high expression (PD-L1 CPS ≥ 5) and low expression (PD-L1 CPS < 5) in GC. The SHAP method can improve the interpretability of ML models, thereby aiding clinicians in comprehending the model and facilitating clinical decision-making.

## Electronic supplementary material

Below is the link to the electronic supplementary material.

Supplementary Material 1

## Data Availability

No datasets were generated or analysed during the current study.

## Abbreviations

CECT: Contrast-enhanced computed tomography
PD-L1: Programmed death ligand 1
ML: Machine learning
CPS: Combined Positive Score
ROI: Regions of interest
LASSO: Least absolute shrinkage and selection operator
ICC: Intraclass correlation coefficient
ROC: Receiver operating characteristic curve
AUC: Areas under the curve
LR: Logistic Regression
NB: NaiveBayes
SVM: Support Vector Machine
KNN: K-Nearest Neighbors
RF: Random Forest
ExtraTrees: Extremely Randomized Trees
XGBoost: Extreme Gradient Boosting
LGBM: Light Gradient Boosting Machine
GBR: Gradient boosting regression
AdaBoost: Adaptive Boosting
MLP: Multilayer Perceptron
NPV: Negative prediction value
PPV: Positive predictive value
