# Supplementary material for: An interpretable machine learning model based on computed tomography radiomics for predicting programmed death ligand 1 expression status in gastric cancer
Source: Cancer Imaging. 2025 Mar 12;25:31. doi: 10.1186/s40644-025-00855-3 (PMC11905525; doi:10.1186/s40644-025-00855-3)
Supplement: Supplementary file 1 — Supplementary Material 1 [file 40644_2025_855_MOESM1_ESM.docx]

**Supplementary A1**

**1. Paraffin section dewaxing and hydration:** Place the tissue section in the oven at 60^℃^ for more than 2h, put it into the Dako Auto stained link 48 automated slide stainer (PD-L1 IHC 22C3 pharmDx assay kit , Agilent Technologies) and choose the dewaxing system (3 times dewaxing in xylene, 10min/times, gradient alcohol hydration, anhydrous ethanol for 5min, 95% alcohol for 5min, 70% alcohol for 5min), and then rinse it once with distilled water, 5min/time.

**2. Antigen repair:** Place the sections on a plastic staining rack in a container containing 0.01MPH6.0 citrate tissue antigen buffer, immerse the sections under the surface of the liquid, and microwave heat them until boiling, remove the container after 15-20min, and allow it to cool naturally to room temperature. Remove the slides from the buffer and rinse them twice with distilled water and then three times with PBS for 3 min/time.

**3. Inactivate endogenous peroxidase:** add peroxidase blocker dropwise to each tissue section, incubate at room temperature for 10min, rinse with PBS for 3 times, 3min each time.

**4. Dropwise addition of antibodies:** remove the PBS, add 1 drop of the configured primary antibody on the section, the working titer of each antibody is: mouse anti-PD-1 (NAT) antibody (1:20), rabbit anti-PD-L1 (SP142) antibody (1:40); then put it into the refrigerator at 4℃ overnight; take it out of the refrigerator the next day and put it into the room to reheat for 30min, rinsed with PBS 3 times, 3min/times. PBS was rinsed three times for 3 min/times. Remove the PBS, add 50ul of MaxVisionTM reagent to each slide, incubate at room temperature for 15min, rinse with PBS 3 times, 3min/time.

**5. DAB colour development:** Remove PBS and add two drops of freshly prepared DAB colour development reagent to each slide. The colour development time was controlled under a light microscope. Terminate the colour development by rinsing with tap water after 3-5min, and rinse for 10min to remove the residual chromogen.

**6. Re-staining:** re-stain the cell nucleus with hematoxylin for 1min, rinse with tap water to return to blue.

**7. Film sealing:** After 70%, 95%, 100% gradient alcohol dehydration for 3min each, xylene transparent for 10min, air-drying by hair dryer, neutral gum sealing.

**8. Immunohistochemistry results determination criteria**: The immunostained tissue sections were scored by two independent pathologists (X.H.X. and Y.L.C. with 10 and 6 years of relevant experience, respectively). Both pathologists were blinded to the patients’ clinical data, and disagreements on CPS assessment were resolved through consensus 2 weeks after individual interpretations. Positive staining was defined as the presence of yellow to tan granules in the cell membrane or cytoplasm. Cases with positive expression of PD-1 and PD-L1 molecules were evaluated by combining the intensity of staining with the percentage of positive cells. The whole section was first observed under a low-power microscope, and five randomly selected high power fields (200×) were selected in the tumour cells and tumour mesenchymal cells, respectively, and 100 cells were counted in each field, and the percentage of positive cells = the number of positive cells (tumour cells, lymphocytes, and macrophages)/the number of observed cells×100%. A CPS score of ≥5 was defined as positive PD-L1 expression, and a CPS score of <5 was classified as negative PD-L1 expression.

**Table S1** The CT scanners and image acquisition protocols of the two centers

| **Parameters** | **Center 1** | **Center 1** | **Center 2** |
| --- | --- | --- | --- |
| **CT version** | GE LightSpeed VCT | GE Revolution HD | United Imaging 780 |
| **kVp** | 120 kVp | 120 kVp | 120 kVp |
| **Tube Current** | 120~480mAs | 120~450mAs | 120~420mAs |
| **Detector collimation** | 64×0.625 | 64×0.625 | 80×0.5 |
| **Rotation time** | 0.37s | 0.37s | 0.3s |
| **Slice thickness** | 5 mm | 5 mm | 5 mm |
| **Image matrix** | 512 × 512 | 512 × 512 | 512 × 512 |
| **Field of view** | 380 mm × 380 mm | 380 mm × 380 mm | 350 mm × 350 mm |

**Table S2** Radiomics features calculated in pyradiomics

| **Statistical descriptors** | **Texture features** |
| --- | --- |
| **First Order Features** | Energy, Entropy, Minimum, 10 Percentile, 90 Percentile, Maximum, Mean, Median, Interquartile Range, Range, Mean Absolute Deviation, Robust Mean Absolute Deviation, Root Mean Squared, Standard Deviation, Skewness, Kurtosis, Variance, Uniformity |
| **Shape Features** | Mesh Surface, Pixel Surface, Perimeter, Perimeter Surface Ratio, Sphericity, Spherical Disproportion, Maximum Diameter, Major Axis Length, Minor Axis Length, Elongation |
| **GLCM** | Autocorrelation, Joint Average, Cluster Prominence, Cluster Shade, Cluster Tendency, Contrast, Correlation, Difference Average, Difference Entropy, Difference Variance, Dissimilarity, Joint Energy, Joint Entropy, IMC1, IMC2, IDM, MCC, IDMN, ID, Inverse Variance, Maximum Probability, Sum Average, Sum Entropy, Sum Squares |
| **GLSZM** | Small Area Emphasis, Large Area Emphasis, Gray Level Non-Uniformity, Gray Level Non-Uniformity Normalized, Size Zone Non-Uniformity, Size Zone Non-Uniformity Normalized, Zone Percentage, Gray Level Variance, Zone Variance, Zone Entropy, Low Gray Level Zone Emphasis, High Gray Level Zone Emphasis, Small Area Low Gray Level Emphasis, Small Area High Gray Level Emphasis, Large Area Low Gray Level Emphasis |
| **GLRLM** | Short Run Emphasis, Long Run Emphasis, Gray Level Non-Uniformity, Gray Level Non-Uniformity Normalized, Run Length Non-Uniformity, Run Length Non-Uniformity Normalized, Run Percentage, Gray Level Variance, Run Variance, Run Entropy, Low Gray Level Run Emphasis, High Gray Level Run Emphasis, Short Run Low Gray Level Emphasis, Short Run High Gray Level Emphasis, Long Run Low Gray Level Emphasis, Long Run High Gray Level Emphasis |
| **NGTDM** | Coarseness, Contrast, Busyness, Complexity, Strength |
| **GLDM** | Small Dependence Emphasis, Large Dependence Emphasis, Gray Level Non-Uniformity, Dependence Non-Uniformity, Dependence Non-Uniformity Normalized, Gray Level Variance, Dependence Variance, Dependence Entropy, Low Gray Level Emphasis, High Gray Level Emphasis, Small Dependence Low Gray Level Emphasis, Small Dependence High Gray Level Emphasis, Large Dependence Low Gray Level Emphasis, Large Dependence High Gray Level Emphasis |
| **Wavelet** | Wavelet transform. Generates all combinations using high-pass and low-pass filters in each of the two dimensions (LL, LH, HH, HL) |

Abbreviations: GLCM, gray level co-occurrence matrix; GLSZM, gray level size zone matrix; GLRLM, gray level run length matrix; NGTDM, neighboring gray tone difference matrix; GLDM, gray level dependence matrix.

**Table S3** Comparison of radiomics features between PD-L1 high expression and low expression groups in the validation and test sets

| **Feature name** | **Radiomics feature value (Z-score normalization)** | | | | | |
| --- | --- | --- | --- | --- | --- | --- |
|  | **Validation set** | |  | **Test set** | |  |
|  | **PD-L1 low expression group**  **(N=31)** | **PD-L1 high**  **Expression group (N=31)** | ***P* value** | **PD-L1 low expression group**  **(N=46)** | **PD-L1 low expression group**  **(N=34)** | ***P* value** |
| wavele_HH_glcm_Idn | 0.15 ± 1.15 | -0.15 ± 0.82 | 0.163 | 0.37 ± 1.03 | -0.50 ± 0.70 | <0.001 |
| wavele_HL_gldm_Dependence-Entropy | 0.37 ± 1.03 | -0.37 ± 0.83 | 0.003 | 0.19 ± 1.00 | -0.26 ± 0.95 | 0.046 |
| wavele_HL_glszm_LargeAreaLowGrayLevelEmphasis | -0.17 ± 1.09 | 0.17 ± 0.88 | 0.008 | -0.28 ± 0.68 | 0.38 ± 1.23 | <0.001 |
| wavele_LL_firstorder_Kurtosis | 0.28 ± 1.09 | -0.28 ± 0.82 | 0.014 | 0.11 ± 1.12 | -0.15 ± 0.81 | 0.057 |
| wavele_LL_firstorder_Minimum | -0.37 ± 0.99 | 0.37 ± 0.87 | 0.002 | -0.09 ± 1.16 | 0.12 ± 0.74 | 0.789 |
| wavele_LL_glcm_Idn | 0.28 ± 0.93 | -0.28 ± 1.00 | 0.026 | 0.29 ± 0.99 | -0.39 ± 0.89 | 0.002 |
| wavele_LL_glcm_JointEntropy | 0.3 2± 0.95 | -0.32 ± 0.96 | 0.012 | 0.18 ± 0.82 | -0.24 ± 1.17 | 0.064 |
| wavele_LL_glcm_Maximum-Probability | -0.28 ± 0.85 | 0.28 ± 1.07 | 0.017 | -0.12 ± 0.79 | 0.16 ± 1.23 | 0.576 |
| wavele_LL_ngtdm_Busyness | -0.45 ± 0.49 | 0.45 ± 1.17 | <0.001 | -0.34 ± 0.44 | 0.46 ± 1.33 | <0.001 |

Note: ^a^Presented as mean ± standard deviation (SD), and compared using an independent *t*-test.

Abbreviations: CPS, combined positive score; glcm, gray level cooccurrence matrix; glszm, gray level size zone matrix; ngtdm, neighboring gray tone difference matrix.

**Table S4** Inter- and intra-observer repeatability for radiomics features included in the radiomics model

| **Radiomics Features** | **Inter-observer agreement** | **Intra-observer agreement** |
| --- | --- | --- |
| wavele_HH_glcm_Idn | 0.852 | 0.831 |
| wavele_HL_gldm_Dependence-Entropy | 0.803 | 0.853 |
| wavele_HL_glszm_LargeAreaLowGrayLevelEmphasis | 0.873 | 0.903 |
| wavele_LL_firstorder_Kurtosis | 0.936 | 0.916 |
| wavele_LL_firstorder_Minimum | 0.928 | 0.933 |
| wavele_LL_glcm_Idn | 0.820 | 0.816 |
| wavele_LL_glcm_JointEntropy | 0.945 | 0.945 |
| wavele_LL_glcm_Maximum-Probability | 0.912 | 0.902 |
| wavele_LL_ngtdm_Busyness | 0.929 | 0.919 |

Note: The intraclass correlation coefficient (ICC) was used to evaluate the inter- and intra-observer repeatability for each radiomics feature.


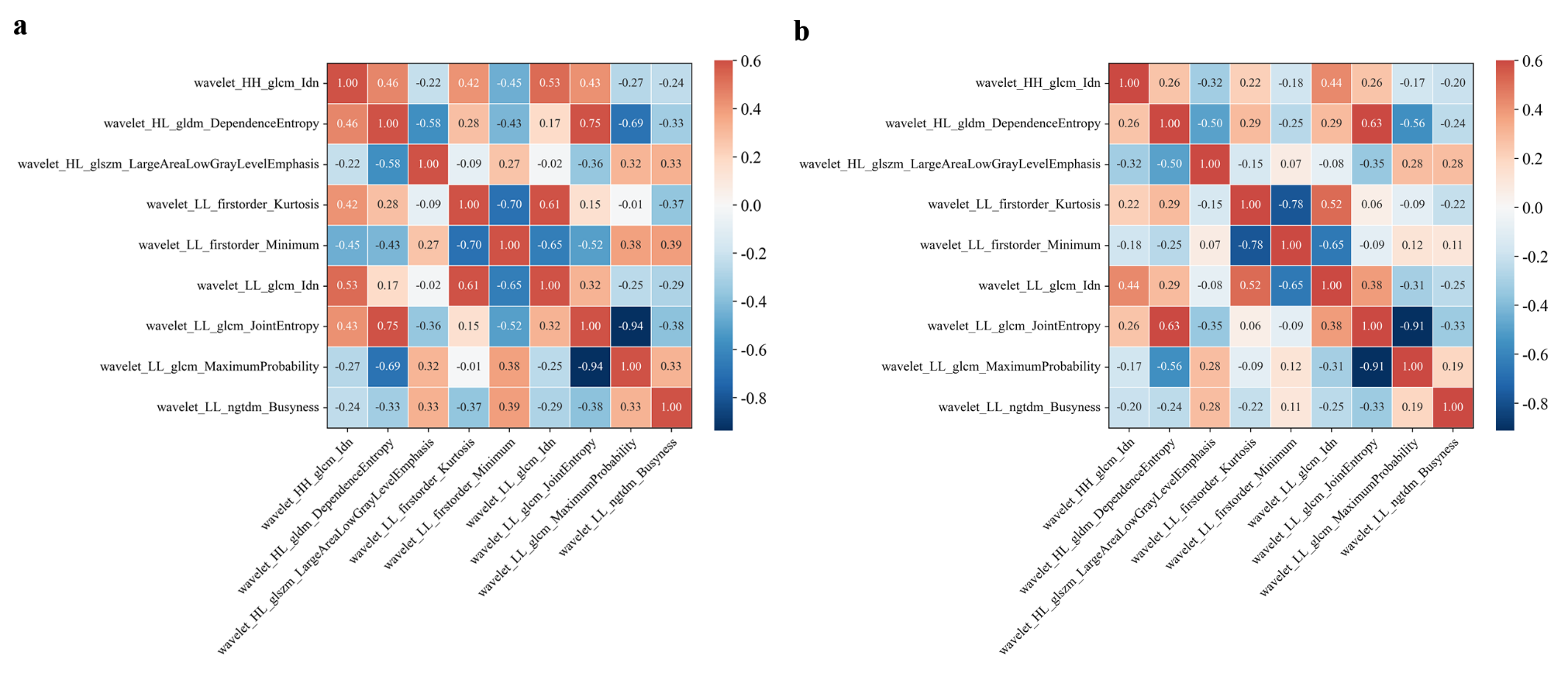


**Fig. S1** Correlation heatmaps of nine radiomics features in the (a) validation and (b) test sets.


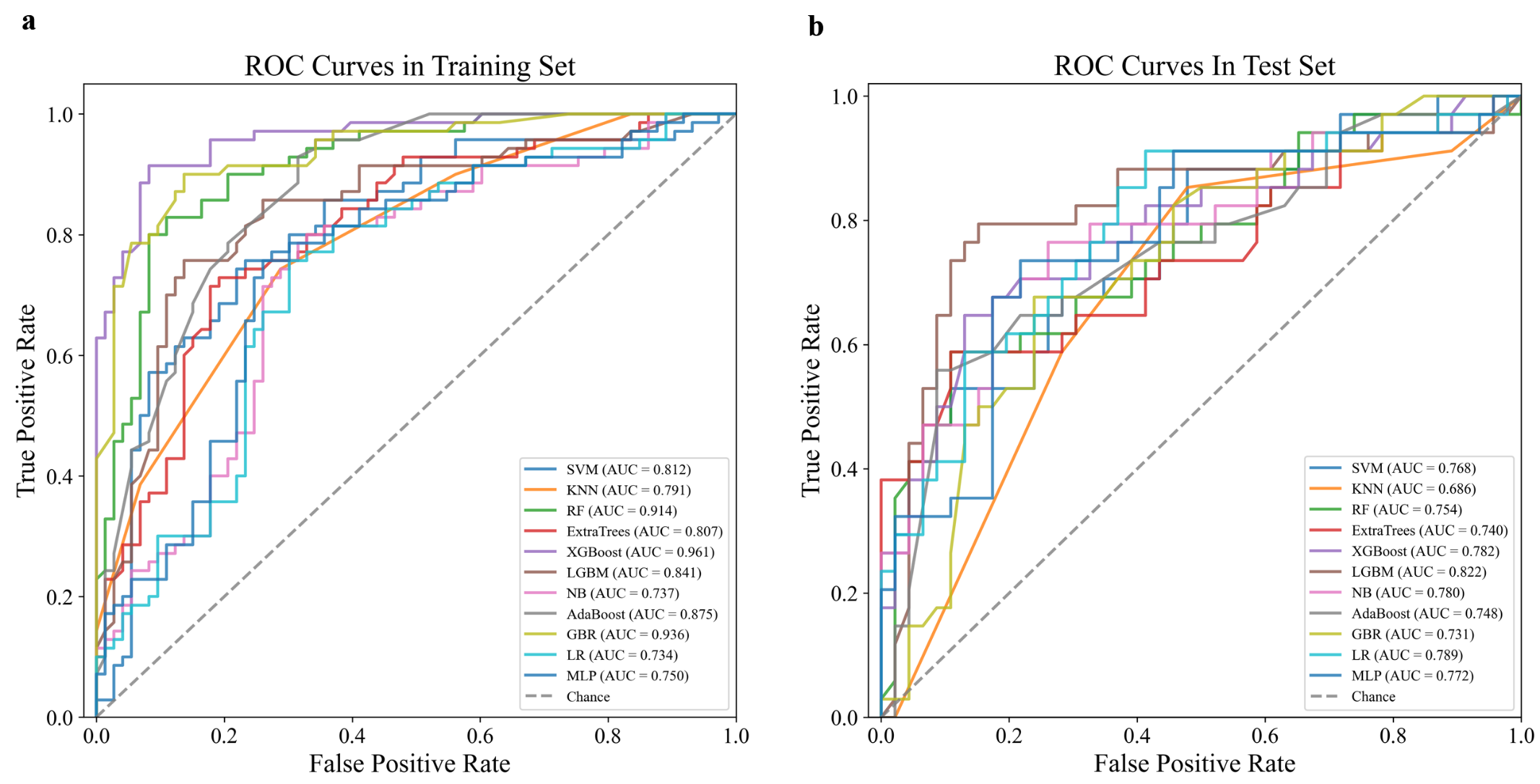


**Fig. S2** ROC curves of 11 ML models in the (a) training and (b) test sets. ML, machine learning.
